# Awareness of disease and Quality of Life of People with Alzheimer's Disease in a Brazilian population

**DOI:** 10.1002/alz70858_099753

**Published:** 2025-12-24

**Authors:** Marcela Moreira Lima Nogueira, Tatiana Belfort, Maria Alice Baptista, Isabel Lacerda, Michelle Brandt, Julia Gaigher, Marcia C. N. Dourado

**Affiliations:** ^1^ Federal University of Rio de Janeiro, Rio de Janeiro, Rio de Janeiro, Brazil; ^2^ Federal University of Rio de Janeiro, Rio de Janeiro, Brazil

## Abstract

**Background:**

Quality of life (QoL) is a complex concept and may be influenced by subjective well‐being. This study aims to comprehend, between different levels of awareness of disease, which clinical characteristics are related to the evaluation of QoL, and to determine whether self‐reported QoL of people with Alzheimer's disease is related to emotional or cognitive/functional domains.

**Method:**

We evaluate QoL of 179 Brazilian people with Alzheimer's disease comparing different levels of awareness of disease (*N* = 32 with preserved awareness; *N* = 88 with mildly impaired awareness; and *N* = 59 with severe deficit/absent awareness). Participants and their carers completed questionnaires about QoL, awareness of disease, cognition, dementia severity, functionality, neuropsychiatric symptoms, and depression.

**Result:**

Individuals with preserved awareness exhibited lower QoL than the group with mildly impairment of awareness. The severe deficit/absent awareness group showed more cognitive impairment (*p* = 0.010), higher presence of neuropsychiatric symptoms (*p* <0.001), more depression severity (*p* = 0.001), lower activity of daily living (*p* <0.001), and higher QoL (*p* <0.001) when compared to other groups of people with Alzheimer's disease. The QoL total was negatively related to the presence of neuropsychiatric symptoms to people with Alzheimer's disease with preserved awareness (*p* = 0.029) and mildly impaired awareness (*p* = 0.004); and related to marital status (*p* = 0.002) for those with severe deficit/absent awareness.

**Conclusion:**

The awareness of disease may inversely influence QoL evaluation. QoL becomes more related to psychosocial factors and emotional well‐being in higher deficits of awareness, while functional aspects are more important in lower deficits of awareness.

